# ﻿Multi-gene phylogenetic and taxonomic contributions to *Xylaria* (Ascomycota) associated with fallen fruits from China

**DOI:** 10.3897/mycokeys.106.124944

**Published:** 2024-06-13

**Authors:** An-Hong Zhu, Zi-Kun Song, Jun-Fang Wang, Hao-Wen Guan, Zhi Qu, Hai-Xia Ma

**Affiliations:** 1 Hainan Key Laboratory of Tropical Microbe Resources, Institute of Tropical Bioscience and Biotechnology, Chinese Academy of Tropical Agricultural Sciences, Haikou 571101, China Institute of Tropical Bioscience and Biotechnology, Chinese Academy of Tropical Agricultural Sciences Haikou China; 2 Haikou Key Laboratory for Protection and Utilization of Edible and Medicinal Fungi, Hainan Institute for Tropical Agricultural Resources, Haikou 571101, China Hainan Institute for Tropical Agricultural Resources Haikou China; 3 School of Ecology and Nature Conservation, Beijing Forestry University, Beijing 100083, China Beijing Forestry University Beijing China; 4 Coconut Research Institute, Chinese Academy of Tropical Agricultural Sciences, Wenchang 571339, China Coconut Research Institute, Chinese Academy of Tropical Agricultural Sciences Wenchang China; 5 College of Plant Protection, Jilin Agricultural University, Changchun 130118, China Jilin Agricultural University Changchun China; 6 School of Life Science, Liaoning University, Shenyang 110036, China Liaoning University Shenyang China; 7 Chongzuo Key Laboratory for Protection and Utilization of Edible and Medicinal Fungi, Fusui 532100, China Chongzuo Key Laboratory for Protection and Utilization of Edible and Medicinal Fungi Fusui China

**Keywords:** Ascomycota, fructicolous fungi, new species, seminicolous fungi, Xylariaceae

## Abstract

Morphological and phylogenetic analyses on samples of *Xylaria* species associated with fallen fruits from China were carried out, and two new species were described, namely *X.aleuriticola* and *X.microcarpa*. *Xylariaaleuriticola* is found on fallen fruits of *Aleuritesmoluccana*, and characterized by stromata dichotomously branched several times with long acute sterile apices, fertile parts roughened with perithecia and tomentose, and ellipsoid to fusiform ascospores. *Xylariamicrocarpa* differs in its very small stromata with dark brown tomentum, light brown ascospores with an inconspicuous straight germ slit, and grows on leguminous pods. The differences between the new species and morphologically similar species are discussed. Phylogenetic analyses on ITS-RPB2-TUB sequences confirmed that the two species are clearly separated from other species of the genus *Xylaria*. *Xylarialiquidambaris* is reported as a new record from China. A key to the *Xylaria* species associated with fallen fruits and seeds reported from China is provided to facilitate future studies of the genus.

## ﻿Introduction

*Xylaria* Hill ex Schrank, with more than 878 epithets listed in Index Fungorum (http://www.indexfungorum.org/Names/Names.asp, accessed on 22 November 2023), was currently the largest genus in the family Xylariaceae (Hsieh et al. 2010; Fournier et al. 2018a). The members of *Xylaria* have a worldwide distribution, but they are highly diverse in the tropics and subtropics (Dennis 1956; Ju and Rogers 1999; Ju and Hsieh 2007; Lodge et al. 2008; Fournier et al. 2011; Wangsawat et al. 2021). Species of *Xylaria* are saprobic, pathogenic, or endophytic and associated with a wide range of host (Rogers 1979a; Vannini et al. 1996; Whalley 1996; Crozier et al. 2006; Thomas et al. 2008; U’Ren et al. 2009; de Vega et al. 2010). According to the substrate in which these fungi grow, the taxa of the genus can be divided into four different ecological types, viz., wood-inhabiting type, termite nests inhabiting type, foliicolous type, and fructicolous/seminicolous type. The *Xylaria* species associated with fallen fruits and seeds preferred to somewhat substrate-specific (Rogers 1979b; Læssøe and Lodge 1994; Ju et al. 2018; Perera et al. 2020).

The generic concept of *Xylaria* was traditionally based on morphological studies (Dennis 1957, 1958; Rogers et al. 1987, 1988; San Martín and Rogers 1989; Fournier 2014; Fournier et al. 2020). In the past two to three decades, molecular phylogenetic analysis was carried out on the family Xylariaceae by using a single-gene to multi-gene (Lee et al. 2000; Bahl et al. 2005; Ju et al. 2004, 2007; Peláez et al. 2008; Hsieh et al. 2010; Læssøe et al. 2013; Wangsawat et al. 2021). Nuclear ribosomal DNA, ITS-5.8S, and protein-coding gene are commonly used for inferring phylogenetic relationships (Tang et al. 2009; Visser et al. 2009). The new genus *Neoxylaria* was segregated from *Xylaria* based on morphological and phylogenetic evidence (Konta et al. 2020). The genus *Xylaria* is quite common in China, however, molecular studies on the *Xylaria* are still poorly used (Teng 1963; Tai 1979; Li and Li 1994; Xu 1999; Zhu and Guo 2011; Ma et al. 2011, 2013). Especially, the phylogenetic relationships inferring from multi-gene between *Xylaria* species associated with fruits and other *Xylaria* species as well as other genera in the Xylariaceae remain unsolved, and the species diversity and geographical distribution in China are unclear.

During the investigation of xylariaceous taxa from China, 18 samples belonging to 3 species of *Xylaria* associated with fruits were collected. Based on morphological and multi-gene phylogenetic evidences, two new species and one new Chinese record are introduced in this study.

## ﻿Materials and methods

### ﻿Sample collection and morphological studies

The studied samples were collected from south China during 2013–2020. The fallen fruits bearing xylariaceous stromata were dried with a SX-770 portable drier of Foshan Taomeihui Electric Appliance Co., Ltd (Guangdong, China), and deposited in the Fungarium of Institute of Tropical Bioscience and Biotechnology, Chinese Academy of Tropical Agricultural Sciences (FCATAS). Macro- and micro-morphological studies were carried out in this study and followed Ma et al. (2022). Stromatal surface and perithecia were observed and measured by using a VHX-600E 3D microscope of the Keyence Corporation (Osaka, Japan). Microscopic observations and examinations were performed by using an Olympus IX73 inverted fluorescence microscope (Tokyo, Japan) and the CellSens Dimensions Software (Olympus, Tokyo, Japan). In presenting ascospore size variation, 5% of measurements were excluded from each end of the range and given in parentheses. The following abbreviations were used: KOH = 5% potassium hydroxide, SDS = 1% sodium dodecyl sulfate, M = mean ascospore length × mean ascospore width, Q = the ration of mean ascospore length / mean ascospore width, n (a/b) = number of ascospores (a) measured from number of specimens (b). Colour terms followed Rayner (1970).

### ﻿DNA extraction, amplification, and sequencing

Total genomic DNA was extracted from dried specimens using a cetyltrimethylammonium bromide (CTAB) rapid extraction kit (Aidlab Biotechnologies, Beijing) following its instruction with some modifications as in Song et al. (2022). Three DNA gene fragments, the internal transcribed spacer (ITS) region, RNA polymerase II subunit (RPB2) gene, and β-tubulin (TUB) were amplified using the primer pairs ITS5/ITS4 (White et al. 1990), fRPB2-5F/fRPB2-7cR (Liu et al. 1999), and T1/T22 (O’ Donnell et al. 1997), respectively. The PCR procedures for the three sequences followed Pan et al. (2022). Newly generated sequences were uploaded on GenBank and listed in Table 1.

**Table 1.** List of taxa used for the phylogenetic reconstruction. GenBank accession numbers, specimen numbers, origin and host are given. Holotype specimens are labelled with HT. Species highlighted in bold were derived from this study. N/A: not available.

Species	Specimen No.	Origin	Host	GenBank Accession Number		
				ITS	RPB2	ß-tubulin
*Amphiroselliniafushanensis*	HAST91111209(HT)	China	dead twigs	GU339496	GQ848339	GQ495950
*A.nigrospora*	HAST91092308(HT)	China	dead twigs	GU322457	GQ848340	GQ49595
*Astrocystisbambusae*	HAST89021904	China	bamboo culms	GU322449	GQ844836	GQ495942
*As.mirabilis*	HAST94070803	China	bamboo culms	GU322448	GQ844835	GQ49594
*Kretzschmariaclavus*	JDR114	French Guiana	wood	EF026126	GQ844789	EF025611
*K.guyanensis*	HAST89062903	China	bark	GU300079	GQ844792	GQ478214
*K.neocaledonica*	HAST94031003	China	bark	GU300078	GQ844788	GQ478213
*Nemaniaabortiva*	BiSH467(HT)	USA	–	GU292816	GQ844768	GQ470219
*N.diffusa*	HAST91020401	China	bark	GU292817	GQ844769	GQ470220
*N.sphaeriostomum*	JDR261	USA	wood	GU292821	GQ844774	GQ470224
*Podosordariamexicana*	WSP176	Mexico	horse dung	GU324762	GQ853039	GQ844840
*P.muli*	WSP167(HT)	Mexico	mule dung	GU324761	GQ853038	GQ844839
*Poroniapileiformis*	WSP88113001(ET)	China	cow dung	GU324760	GQ853037	GQ502720
*Roselliniamerrillii*	HAST89112601	China	bark	GU300071	GQ844781	GQ470229
*R.sanctacruciana*	HAST90072903	China	fronds of *Arengaengleri*	GU292824	GQ844777	GQ470227
*Xylariaadscendens*	HAST570	Guadeloupe	wood	GU300101	GQ844817	GQ487708
*X.aethiopica*	YMJ1136	Ethiopia	pods of *Millettiaferruginea*	MH790445	MH785222	MH785221
***X.aleuriticola***	**FCATAS858(HT)**	**China**	**fruits of *Aleuritesmoluccana***	**MZ648856**	**MZ707101**	**MZ695778**
***X.aleuriticola***	**FCATAS859**	**China**	**fruits of *Aleuritesmoluccana***	**MZ648857**	**MZ707102**	**MZ695779**
***X.aleuriticola***	**FCATAS862**	**China**	**fruits of *Aleuritesmoluccana***	**MZ648858**	**N/A**	**MZ695780**
***X.aleuriticola***	**FCATAS863**	**China**	**fruits of *Aleuritesmoluccana***	**MZ648859**	**N/A**	**MZ695781**
***X.aleuriticola***	**FCATAS864**	**China**	**fruits of *Aleuritesmoluccana***	**MZ648860**	**MZ707103**	**N/A**
*X.allantoidea*	HAST94042903	China	trunk	GU324743	GQ848356	GQ502692
*X.amphithele*	HAST529	Guadeloupe	dead leaves	GU300083	GQ844796	GQ478218
*X.apoda*	HAST90080804	China	bark	GU322437	GQ844823	GQ495930
*X.arbuscula*	HAST89041211	China	bark	GU300090	GQ844805	GQ478226
*X.atrosphaerica*	HAST91111214	China	bark	GU322459	GQ848342	GQ495953
*X.berteri*	HAST90112623	China	wood	GU324749	GQ848362	AY951763
*X.brunneovinosa*	HAST720(HT)	China	ground of bamboo plantation	EU179862	GQ853023	GQ502706
*X.cirrata*	HAST664(ET)	China	ground of vegetable farm	EU179863	GQ853024	GQ502707
*X.cranioides*	HAST226	China	wood	GU300075	GQ844785	GQ478210
*X.cubensis*	JDR860	USA	wood	GU991523	GQ848365	GQ502700
*X.culleniae*	JDR189	Thailand	pod	GU322442	GQ844829	GQ495935
*X.curta*	HAST92092022	China	bark	GU322443	GQ844830	GQ495936
*X.digitata*	HAST919	Ukraine	wood	GU322456	GQ848338	GQ495949
*X.enterogena*	HAST785	French Guiana	wood	GU324736	GQ848349	GQ502685
*X.escharoidea*	HAST658(ET)	China	ground of mango orchard	EU179864	GQ853026	GQ502709
*X.fabacearum*	MFLU16-1061(HT)	Thailand	seed pods of Fabaceae	NR171104	MT212202	MT212220
*X.fabaceicola*	MFLU16-1072(HT)	Thailand	seed pods of Fabaceae	NR171103	MT212201	MT212219
***Xylaria* sp.**	**FCATAS917**	**China**	**pericarps of *Faguslongipetiolata***	**MZ621171**	**MZ707122**	**MZ695801**
*X.feejeensis*	HAST92092013	China	bark	GU322454	GQ848336	GQ495947
*X.fimbriata*	HAST491	Martinique	termite nest	GU324753	GQ853022	GQ502705
*X.fissilis*	HAST367	Martinique	bark	GU300073	GQ844783	GQ470231
*X.frustulosa*	HAST92092010	China	bark	GU322451	GQ844838	GQ495944
X.cf.glebulosa	HAST431	Martinique	Fruits of *Swieteniamacrophylla*	GU322462	GQ848345	GQ495956
*X.globosa*	HAST775	Guadeloupe	bark	GU324735	GQ848348	GQ502684
*X.grammica*	HAST479	China	wood	GU300097	GQ844813	GQ487704
*X.griseosepiacea*	HAST641(HT)	China	ground of mango orchard	EU179865	GQ853031	GQ502714
*X.haemorrhoidalis*	HAST89041207	China	bark	GU322464	GQ848347	GQ502683
*X.hedyosmicola*	FCATAS856(HT)	China	leaves of *Hedyosmumorientale*	MZ227121	MZ221183	MZ683407
*X.hypoxylon*	HAST95082001	China	wood	GU300095	GQ844811	GQ487703
*X.intracolorata*	HAST90080402	China	bark	GU324741	GQ848354	GQ502690
*X.ianthinovelutina*	HAST553	Martinique	fruit of *Swieteniamacrophylla*	GU322441	GQ844828	GQ495934
*X.intraflava*	HAST725(HT)	China	ground of bamboo plantation	EU179866	GQ853035	GQ502718
*X.juruensis*	HAST92042501	China	*Arengaengleri*	GU322439	GQ844825	GQ495932
*X.laevis*	HAST95072910	China	bark	GU324747	GQ848360	GQ502696
*X.lindericola*	FCATAS852	China	leaves of *Linderarobusta*	MZ005635	MZ031982	MZ031978
*X.liquidambaris*	HAST93090701	China	fruits of *Liquidambarformosana*	GU300094	GQ844810	GQ487702
***X.liquidambaris***	**FCATAS872**	**China**	**fruits of *Liquidambarformosana***	**MZ620273**	**MZ707106**	**N/A**
***X.liquidambaris***	**FCATAS874**	**China**	**fruits of *Liquidambarformosana***	**MZ620275**	**MZ707107**	**MZ695775**
***X.liquidambaris***	**FCATAS877**	**China**	**fruits of *Liquidambarformosana***	**MZ620276**	**MZ707109**	**N/A**
***X.liquidambaris***	**FCATAS879**	**China**	**fruits of *Liquidambarformosana***	**MZ620278**	**MZ707110**	**N/A**
*X.meliacearum*	JDR148	Puerto Rico	petioles and infructescence of *Guareaguidonia*	GU300084	GQ844797	GQ478219
***X.microcarpa***	**FCATAS883(HT)**	**China**	**pods of legume**	**MZ648823**	**MZ707111**	**MZ695776**
***X.microcarpa***	**FCATAS885**	**China**	**pods of legume**	**MZ648824**	**N/A**	**MZ695777**
*X.microceras*	HAST414	Guadeloupe	wood	GU300086	GQ844799	GQ478221
*X.montagnei*	HAST495	Martinique	wood	GU322455	GQ848337	GQ495948
*X.multiplex*	JDR259	USA	wood	GU300099	GQ844815	GQ487706
*X.muscula*	HAST520	Guadeloupe	dead branch	GU300087	GQ844800	GQ478222
*X.nigripes*	HAST653	China	ground of mango orchard	GU324755	GQ853027	GQ502710
*X.ochraceostroma*	HAST401(HT)	China	ground of mango orchard	EU179869	GQ853034	GQ502717
*X.oligotoma*	HAST784	French Guiana	wood	GU300092	GQ844808	GQ487700
*X.ophiopoda*	HAST93082805	China	bark	GU322461	GQ848344	GQ495955
*X.oxyacanthae*	JDR859	USA	seeds of *Crataegusmonogyna*	GU322434	GQ844820	GQ495927
*X.palmicola*	PDD604	New Zealand	fruits of palm	GU322436	GQ844822	GQ495929
*X.papulis*	HAST89021903	China	wood	GU300100	GQ844816	GQ487707
*X.phyllocharis*	HAST528	Guadeloupe	dead leaves	GU322445	GQ844832	GQ495938
*X.plebeja*	HAST91122401	China	trunk of *Machiluszuihoensis*	GU324740	GQ848353	GQ502689
*X.polymorpha*	JDR1012	USA	wood	GU322460	GQ848343	GQ495954
*X.polysporicola*	FCATAS848(HT)	China	leaves of *Polysporahainanensis*	MZ005592	MZ031980	MZ031976
*X.reevesiae*	HAST90071609(HT)	China	fruits of *Reevesiaformosana*	GU322435	GQ844821	GQ495928
*X.regalis*	HAST920	India	log of *Ficusracemosa*	GU324745	GQ848358	GQ502694
*X.rogersii*	FCATAS915(HT)	China	fruits of *Magnolia* sp.	MZ648827	MZ707121	MZ695800
*X.schimicola*	FCATAS896(HT)	China	fruits of *Schimanoronhae*	MZ648850	MZ707114	MZ695787
*X.schweinitzii*	HAST92092023	China	bark	GU322463	GQ848346	GQ495957
*X.scruposa*	HAST497	Martinique	wood	GU322458	GQ848341	GQ495952
*X.sicula*	HAST90071613	China	fallen leaves	GU300081	GQ844794	GQ478216
*Xylaria* sp. 6	JDR258	USA	leaves of *Tibouchinasemidecandra*	GU300082	GQ844795	GQ478217
*X.striata*	HAST304	China	branch of *Punicagranatum*	GU300089	GQ844803	GQ478224
*X.telfairii*	HAST90081901	China	bark	GU324738	GQ848351	GQ502687
*X.theaceicola*	FCATAS903(HT)	China	fruits of *Schimavillosa*	MZ648848	MZ707115	MZ695788
*X.tuberoides*	HAST475	Martinique	wood	GU300074	GQ844784	GQ478209
*X.venustula*	HAST 88113002	China	bark	GU300091	GQ844807	GQ487699
*X.vivantii*	HAST519(HT)	Martinique	fruits of *Magnolia* sp.	GU322438	GQ844824	GQ495931
*X.wallichii*	FCATAS923(HT)	China	fruits of *Schimawallichii*	MZ648861	MZ707118	MZ695793

### ﻿Phylogenetic analyses

*Xylaria* species associated with fallen fruits and seeds were subjected to phylogenetic analyses in other various species of *Xylaria* and closely related genera including *Amphirosellinia*, *Astrocystis*, *Kretzschmaria*, *Nemania*, *Podosordaria*, and *Rosellinia* (Table 1). *Poroniapileiformis* (Berk.) Fr. was selected as an outgroup (Wangsawat et al. 2021; Ma et al. 2022).

The sequences of ITS, RPB2 and TUB2 were aligned individually using the online MAFFT tool (http://mafft.cbrc.jp/alignment/server/index.html), and improved manually using BioEdit 7.0.5.3 (Hall 1999) and ClustalX 1.83 (Thompson et al. 1997). The individual gene data sets were concatenated using the MEGA 6.0 (Tamura et al. 2011). The concatenated data set of ITS, RPB2 and TUB (ITS-RPB2-TUB) data set of studied species were carried out using Bayesian inference (BI) and maximum likelihood (ML) analyses. Maximum likelihood (ML) analysis was conducted by raxmlGUI 2.0 using rapid bootstrapping with 1000 replicates, and GTRGAMMA+G as a substitution model (Felsenstein 1981). Bayesian inference (BI) analysis was performed in MrBayes 3.2.6 with jModelTest 2 conducting model discrimination (Huelsenbeck and Ronquist 2001). Six simultaneous Markov chains were run from random starting trees for 1 million generations, and trees were sampled every 1000^th^ generations. The first 25% of sampled trees were discarded as burn-in, and the remaining were used to calculate the posterior probability (PP) of each branch (Larget and Simon 1999). The combined alignment and phylogenetic tree were deposited in Figshare (https://figshare.com/s/e1c181f1e3a56164ecc3).

## ﻿Results

### ﻿Molecular phylogeny

**Figure 1.**
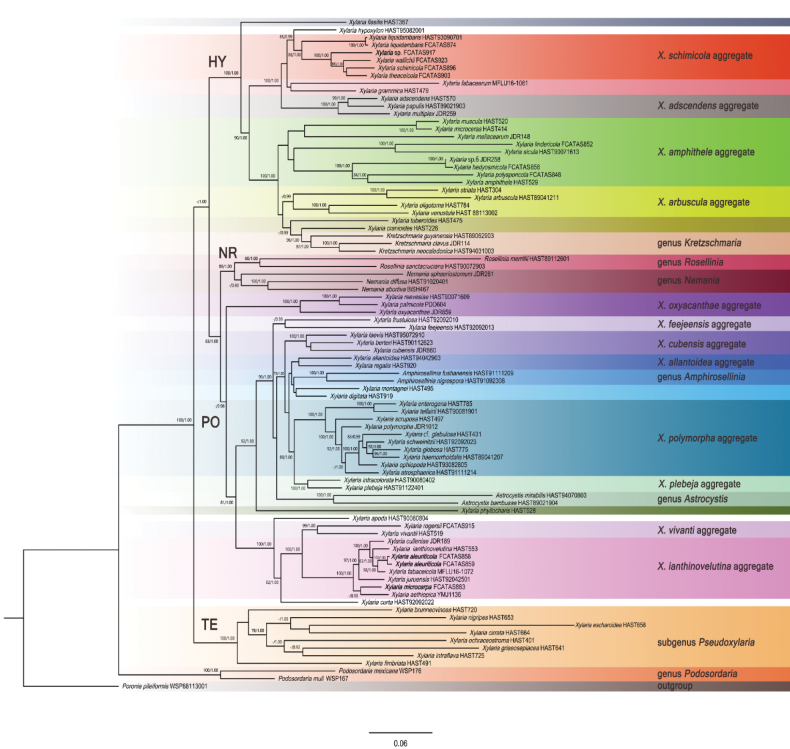
Phylogenetic tree of *Xylaria* based on the multigene alignment of ITS-RPB2-TUB2 in the ML tree. ML bootstrap support (BS) ≥ 75% and Bayesian posterior probabilities (PP) ≥ 0.90 are given at the nodes in this order. New species in this study are indicated in bold.

Eighteen *Xylaria* species associated with fallen fruits and seeds were subjected to phylogenetic analyses based on ITS-RPB2-TUB dataset in Xylariaceae. The BI and ML analyses generated highly similar topologies, the ML tree is presented with bootstrap values ≥ 75% and Bayesian posterior probabilities ≥ 0.90 respectively (Fig. 1).

In the phylogenetic tree (Fig. 1), the genus *Podosordaria* separated from other genera, *Amphirosellinia*, *Astrocystis*, *Kretzschmaria*, *Nemania*, and *Rosellinia* were nested within *Xylaria* clade. All *Xylaria* species associated with fallen fruits and seeds were distributed within clade HY or clade PO as shown in Hsieh et al. (2010) and Ma et al. (2022). In HY clade, a *Xylaria* species on fruits of *Faguslongipetiolata* and four known *Xylaria* species associated with pericarps of fruits, including *X.schimicola* Hai X. Ma & Yu Li, *X.theaceicola* Hai X. Ma & Yu Li, *X.wallichii* Hai X. Ma & Yu Li and *X.liquidambaris* J.D. Rogers, Y.M. Ju & F. San Martín, formed a subclade with high support values (BS = 88, PP = 1.00). In the PO clade, the new species *X.aleuriticola* on fruits of *Aleuritesmoluccana* and *X.microcarpa* on pods grouped with six fructicolous *Xylaria* species including *X.aethiopica* J. Fourn., Y.M. Ju, H.M. Hsieh & U. Lindem., *X.ianthinovelutina* (Mont.) Fr., *X.culleniae* Berk. & Broome, *X.fabaceicola* R.H. Perera, E.B.G. Jones & K.D. Hyde, *X.vivantii* Y.M. Ju, J.D. Rogers, J. Fourn. & H.M. Hsieh, *X.rogersii* Hai X. Ma & Yu Li, and *X.juruensis* Henn. on *Arengaengleri* in a subclade with high support values (BS = 100, PP = 1.00).

### ﻿Taxonomy

#### ﻿ Xylaria aleuriticola

Taxon classificationFungiXylarialesXylariaceae

Hai X. Ma, A.H. Zhu & Yu Li sp. nov.

3EF628DE-D837-5F89-9FE8-E2FEBA815B07

840908

Fig. 2

##### Type.

**China**. Yunnan Province, Jinghong City, Xishuangbanna Primeval Forest Park, on buried fruits of *Aleuritesmoluccana* (L.) Willd (Euphorbiaceae), 22 October 2013, Ma HaiXia, FCATAS 858 (Col. 11).

##### Etymology.

*Aleuriticola* (Lat.): referring to the host which the fungus inhabits.

##### Teleomorph.

Stromata upright or prostrate, solitary to often densely clustered, dichotomously branched several times, or unbranched infrequently, 2–11 cm total height, long-stipitate; fertile parts 7–30 mm high × 1.0–2.5 mm broad, narrowly fusiform to cylindrical, often flattened, with acute sterile apices up to 8 mm long, strongly nodulose, particularly tomentose; stipes 12–90 mm high × 0.7–2.6 mm broad, terete to rarely flattened, most often contorted, usually ill-defined, with conspicuously tomentose, arising from a slightly enlarged pannose base; surface roughened with perithecial mounds and tomentose except for stromatal apices, black brown to black; interior white to cream, tan at center, solid, woody. Perithecia subglobose, 300–500 µm. Ostioles conic-papillate. Asci eight-spored arranged in uniseriate manner, cylindrical, long-stipitate, (90–)110–135(–150) µm total length, the spore-bearing parts (55–)60–70(–75) µm long × (5.5–)6.0–7.0(–7.5) µm broad, the stipes 30–70 µm long, with apical ring bluing in Melzer’s reagent, urn-shaped, 2.0–2.8 µm high × 1.0–1.8 µm diam. Ascospores brown to dark brown, unicellular, ellipsoid to fusiform, inequilateral, with narrowly rounded ends, occasionally one end slightly pinched, smooth, (7.1–)7.5–9.5(–10.5) × (3–)3.5–4(–4.5) µm (M = 8.1 × 3.6 µm, Q = 2.3, n = 60/2), with a conspicuous straight germ slit spore-length or slightly less than spore-length, lacking a hyaline sheath or appendages visible in india ink or 1% SDS.

**Figure 2.**
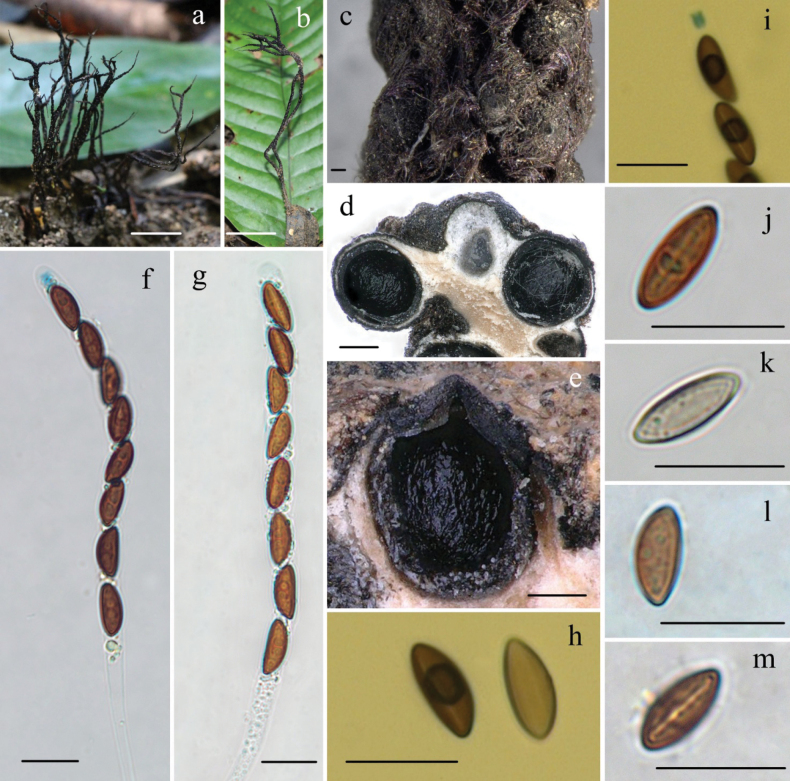
*Xylariaaleuriticola* (FCATAS858, holotype) **a, b** stromata on fallen fruits **c** stromatal surface **d, e** section through stroma, showing perithecia **f** asci in Melzer’s reagent **g** asci in water **h** ascospores in Melzer’s reagent **i** ascal apical ring in Melzer’s reagent **j, k** ascospore in 1% SDS **l** ascospore in India ink **m** ascospore with germ slit in India ink. Scale bars: 2 cm (**a, b**); 100 µm (**c, e**); 200 µm (**d**); 10 µm (**f–m**).

##### Additional specimen examined.

**China**. Yunnan Province, Jinghong City, Xishuangbanna Primeval Forest Park, on buried fruits of *Aleuritesmoluccana* (Euphorbiaceae), 22 October 2013, Ma HaiXia, FCATAS 859 (Col. 23); 22 January 2015, Ma Haixia, FCATAS 862 (Col. 231), FCATAS 863 (Col. 232), FCATAS 864 (Col. 238), FCATAS 865 (COL. 270).

##### Notes.

*Xylariaaleuriticola*, associated with the pericarps of *A.moluccana* (Euphorbiaceae), is characterized by stromata dichotomously branched several times with long acute sterile apices, fertile parts roughened with perithecia and tomentose, and tomentose stipes. It is similar to *X.culleniae* Berk. & Broome by having dichotomously branched stromata and ascospores dimensions, but the latter species branches dichotomously only once in fertile parts, ascospores surrounded with a hyaline sheath and non-cellular appendages, and grows on capsules of *Culleniaexcelsa* (Malvaceae) (Rogers et al. 1988; Ju et al. 2018). *Xylariaeuphorbiicola* Rehm was described on fruits of *Euphorbia* (Euphorbiaceae) from Brazil, but it has unbranched stromata, lacking perithecial mounds, overlain with a brown striped outermost layer, and smaller discoid apical ring 1µm high × 1.5–2 µm broad (Ju et al. 2018). *Xylariaianthinovelutina* somewhat resembles *X.aleuriticola* in stromatal morphology, but it has stronger stromata, larger ascospores (9–)9.5–11(–12) × (3.5–)4–4.5(–5) µm (M = 10.3 × 4.0 µm), and often associated with leguminous pods (Dennis 1956, 1957; Ju et al. 2018), while stromata of the new speices has sharper and longer sterile apices, more forked. *Xylarialuzonensis* Henn. differs from *X.aleuriticola* by its smaller stromata (1.5–3 cm long × 0.5–1 mm diam), smaller perithecia (200–300 µm diam), slightly smaller apical ring (1–1.5 µm high × 1.5 µm broad), light brown ascospores, and grows on pod of *Bauhiniacumingiana* (Fabaceae) (Ju et al. 2018). *Xylariaapeibae* Mont. is close to *X.aleuriticola* in stromatal morphology, from which it differs mainly by having smaller stromata 4 cm long × 0.8–1.5 mm diam, light brown and larger ascospores (9.5–)10–12(–13) × (3–)3.5–4(–4.5) µm (M = 11.0 × 3.7 µm), and grows on fruits of *Apeiba* species (Tiliaceae) (Ju et al. 2018). In the phylogenetic analysis (Fig. 1), *X.aleuriticola* clustered together with high support values (BS = 98, PP = 1.00) with *X.fabaceicola*, but the latter species is distinguished by its smaller stromata 13–25 mm long, pale brown to brown ascospores with a hyaline sheath and appendages, and the fact that it grows on decaying pods of Fabaceae (Perera et al. 2020).

#### ﻿ Xylaria microcarpa

Taxon classificationFungiXylarialesXylariaceae

Hai X. Ma & Yu Li sp. nov.

49706906-6F55-5266-989F-5646D60F227F

840911

Fig. 3

##### Type.

**China**. Yunnan Province, Xishuangbanna Prefecture, Dadugang Town, Guanping Village, on legume pods, 21 January 2015, Haixia Ma, FCATAS 883 (Col. 233).

##### Etymology.

*Microcarpa* (Lat.): referring to its stroma that it is very small.

##### Teleomorph.

Stromata upright or prostrate, often densely gregarious in large groups, unbranched, cylindrical to filiform, with acute sterile apices, on tomentose stipes, 3.5–9 mm total height; fertile parts 2–6 mm high × 0.6–1.5 mm broad, filiform to cylindrical, brown tomentose dense or sparse, nodulose with perithecial contours exposed; stipes 1.5–4 mm high × 0.3–0.5 mm broad, terete, with conspicuously dark brown tomentose, arising from slighly enlarged base; surface black, interior light yellow, solid, woody. Perithecia subglobose, 300–500 µm. Ostioles conic-papillate. Asci eight-spored arranged in uniseriate manner, cylindrical, long-stipitate, (96–)105–125(–140) µm total length, the spore-bearing parts (56–)60–70(–75) µm long ×(6.0–)6.4–7.1(–7.6) µm broad, the stipes 30–56 µm long, with apical ring bluing in Melzer’s reagent, tubular or urn-shaped, 1.5–2.5 (–2.9) µm high × 1.4–1.8 µm diam. Ascospores light brown, unicellular, ellipsoid-inequilateral, with narrowly rounded ends, sometimes with pinched on one end, smooth, (9.5–)10–11(–11.5) ×(4.5–) 5–6(–6.2) µm (M = 10.5 × 5.5 µm, Q = 1.9, n = 60/2), with a inconspicuous straight germ slit almost spore-length, lacking a sheath or appendages visible in india ink or 1% SDS.

**Figure 3.**
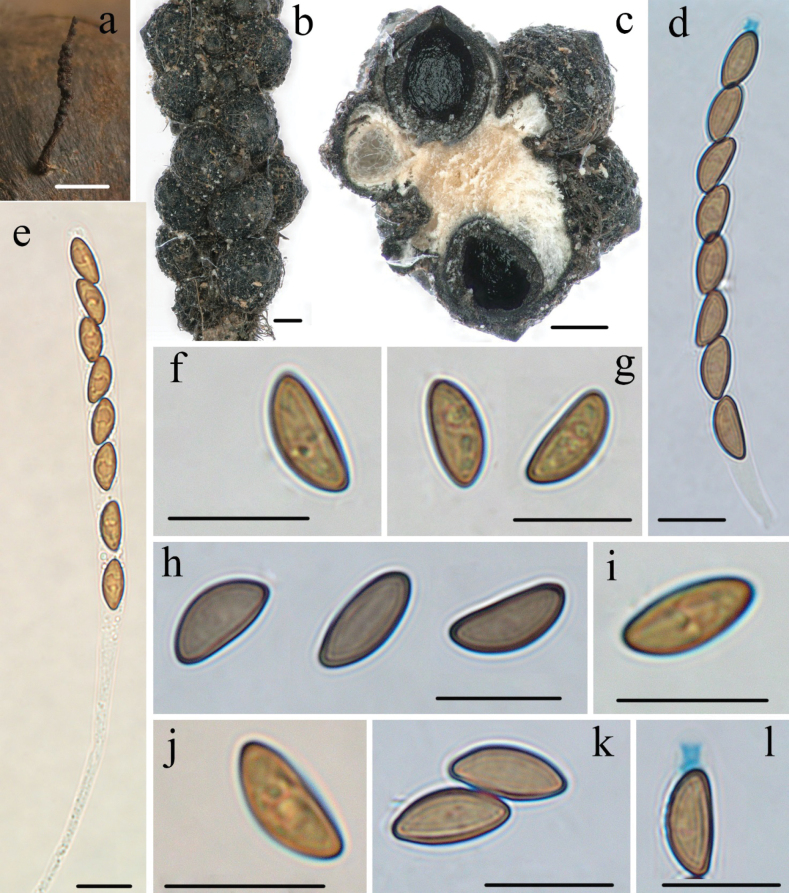
*Xylariamicrocarpa* (FCATAS883, holotype) **a** stroma on fallen pod **b** stromatal surface **c** section through stroma, showing perithecia **d** asci with ascal apical ring in Melzer’s reagent **e** asci in India ink **f, g** ascospores in water **h** ascospores in Melzer’s reagent **i** ascospore with germ slit in India ink **j** ascospore in India ink **k** ascospores in Melzer’s reagent **l** ascal apical ring in Melzer’s reagent. Scale bars: 0.3 mm (**a**); 200 µm (**b, c**); 10 µm (**d–l**).

##### Additional specimen examined.

**China**. Yunnan Province, Xishuangbanna Prefecture, Xishuangbanna Tropical Botanical Garden, on legume pods, 20 January 2015, Haixia Ma, FCATAS 885 (Col. 239).

##### Notes.

*Xylariamicrocarpa* is characterized by very small stromata growing in groups, overlain with a dark brown tomentum, ascospores light brown with an inconspicuous straight germ slit, lacking a sheath or appendages, and grows on leguminous pods. The new species resembles *X.fabacearum* R.H. Perera, E.B.G. Jones & K.D. Hyde by sharing small stromata and ascospores length dimensions, but differs from the latter species in having stromata branched sometimes, stromatal surface without tomentose, brown to dark brown ascospores with conspicuous straight germ slit (Perera et al. 2020). *Xylarialuzonensis* on *Bauhiniacumingiana* (Fabaceae) differs from *X.microcarpa* by having branched and larger stromata, smaller perithecia, and smaller ascospores (8–)8.5–9.5(–10) × 3–3.5(–4) µm (M = 8.9 × 3.4 µm) (Ju et al. 2018). *Xylariamicrocarpa* is somewhat similar to *X.ianthinovelutina* and *X.culleniae* in stromatal surface with tomentum and grow on leguminous pods, but the later two taxa differ in larger stromata, ascospores with a straight germ slit slightly less than spore-length, surrounded with a hyaline sheath and non-cellular appendages (Ju et al. 2018). The phylogenetic tree showed that *Xylariamicrocarpa* and *X.aethiopica* J. Fourn., Y.M. Ju, H.M. Hsieh & U. Lindem are sister taxa with a strong supported branch in BI tree (BS=0.98), but *X.aethiopica* is distinct morphologically with larger stromata 15–30 mm total height, brown to dark brown and slightly larger ascospores (9.7–)11–13(–13.5) × (3.5–)3.8–4.5(–4.9) µm (M = 11.9 × 4.1 µm) with a conspicuous straight germ and appendages, and grows on fallen woody pods of *Millettiaferruginea* (Fabaceae) (Fournier et al. 2018b).

#### ﻿ Xylaria liquidambaris

Taxon classificationFungiXylarialesXylariaceae

J.D. Rogers, Y.M. Ju & F. San Martín, Sydowia 54(1): 92. 2002

CEA34919-8325-5A45-A08A-061AECABFB7A

Fig. 4

##### Teleomorph.

Stromata upright, solitary or sometimes clustered, unbranched or occasionally branched, 1.2–8.0 cm total height; fertile parts 6–25 mm high × 1.5–5.0 mm broad, cylindrical with acute sterile apices, at times longitudinally furrowed, with wrinkles isolating somewhat prominent perithecia; stipes 6–55 mm high × 1.0–2.5 mm broad, glabrous to pubescent arising from a pannose base; surface dark brown to black, interior white, with dark brown to black a circle, and white at center. Texture solid, soft, woody. Perithecia subglobose, 250–400 µm. Ostioles conic-papillate. Asci eight-spored arranged in uniseriate manner, cylindrical, long-stipitate, (110–)125–145(–165) µm total length, the spore-bearing parts (80–)90–105(–115) µm long × (6–)7–8(–8.5) µm broad, the stipes 30–60 µm long, with apical ring bluing in Melzer’s reagent, inverted hap-shaped to more or less rectangular, 2.5–3.5 µm high × 2.0–2.5 µm diam. Ascospores brown, unicellular, ellipsoid-inequilateral with narrowly to broadly rounded ends, smooth, (12.5–)13–14(–15) × (4.8–)5.5–6.5(–6.8) µm (M = 13.5 × 6.1 µm, Q = 2.2, n = 90/3), with spiraling germ slit, lacking a sheath or appendages in india ink or 1% SDS.

##### Specimens examined.

**China**. Guangdong Province, Chebaling Nature Reserve, on fruits of *Liquidambarformosana*, 26 June 2010, Ma Haixia, Col. 10062607; Fengkai County, Heishiding Nature Reserve, on fruits of *L.formosana*, 2 July 2010, Ma Haixia, Col. 10070206; Jiangxi Province, Guanshan Nature Reserve, on fruits of *L.formosana*, 21 June 2013, Ma Haixia, FCATAS 873 (Col. 16); Fuzhou City, Tang Xianzu Museum, on fruits of *L.formosana*, 17 June 2013, Ma Haixia, FCATAS 877 (Col. 36); Anyuan County, Sanbai Mountain Nature Reserve, on fruits of *L.formosana*, 15 August 2016, Ma Haixia, FCATAS 878 (Col. O37); Zhejiang Province, Tianmu Mountain Nature Reserve, on fruits of *L.formosana*, 6 August 2013, Ma Haixia, FCATAS 872 (Col. 10); Gutian Mountain Nature Reserve, on fruits of *L.formosana*, 13 August 2013, Ma Haixia, FCATAS 496 (Col. 29); Anhui Province, Huangshan City, Qiman County, Guniujiang Nature Reserve, on fruits of *L.formosana*, 8 August 2013, Ma Haixia, FCATAS 874 (Col. 19); Huangshan Nature Reserve, on fruits of *L.formosana*, 27 June 2019, Ma Haixia, FCATAS 879 (Col. P6); Hainan Province, Diaoluoshan Nature Reserve, on fruits of *L.formosana*, 31 December 2020, Ma Haixia, FCATAS 880 (Col. Z211).

**Figure 4.**
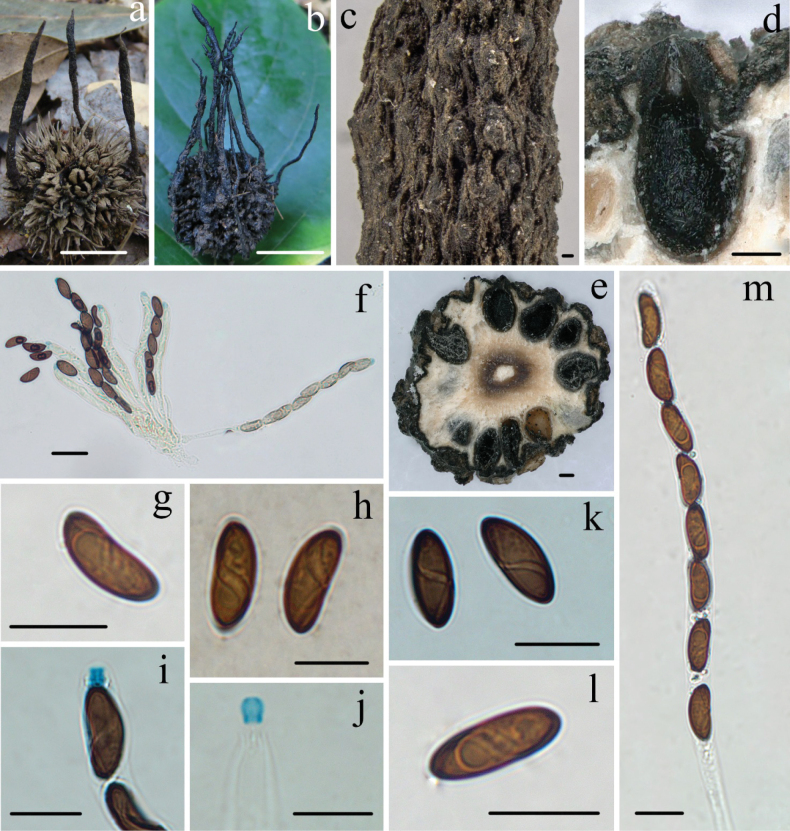
*Xylarialiquidambaris* (**a** from Col.10062607 **b–m** from FCATAS874) **a, b** stromata on fallen fruits **c** stromatal surface **d, e** section through stroma, showing perithecia **f** asci in Melzer’s reagent **g, l** ascospore in water **h** ascospores with germ slit in India ink **i, j** ascal apical ring in Melzer’s reagent **k** ascospores with germ slit in Melzer’s reagent **m** asci in water. Scale bars: 1.5 cm (**a, b**); 100 µm (**c, d, e**); 20 µm (**f**); 10 µm (**g–m**).

##### Notes.

*Xylarialiquidambaris* was originally described by Rogers et al. (2002) from USA, and has high specificity to fruits of *Liquidambar* (Altingiaceae). It is characterized by unbranched stromata with acute sterile apex, embedded to slightly prominent perithecia with longitudinal striations, brown ascospores with long spiraling germ slit (Rogers et al. 2002). These Chinese materials well fit the descriptions and illustrations of *X.liquidambaris* by Rogers et al. (2002).

## ﻿Discussion

In the present study, two new *Xylaria* species associated with fallen fruits were described and compared with closely related species based on morphological and molecular data. In addition, *X.liquidambaris* has been reported from China for the first time. We included eighteen *Xylaria* species on fallen fruits and seeds in the phylogenetic trees based on a combined ITS-RPB2-TUB2 dataset. The phylogenetic analyses showed that seventeen species are mainly distributed in three different subclades, while Xylariacf.gleculosa clustered with *Xylaria* species on wood, which is consistent with the previous studies (Hsieh et al. 2010; Perera et al. 2020; Ma et al. 2022).

By inclusion of the two new species we described here, thirty-seven species on fallen fruits and seeds are now recognized in the genus *Xylaria* (Rogers 1979b; Stowell and Rogers 1983; Rogers et al. 2002; Pande and Waingankar 2004; Rönsch et al. 2010; Hsieh et al. 2010; Dillon et al. 2018; Ju et al. 2018; Fournier et al. 2018b; Perera et al. 2020; Ma et al. 2022). Compared to the number of *Xylaria* species on fruits and seeds, the available sequences of these species in NCBI are relatively fewer. Most species in this group are lacking DNA sequences, and some species only have one or two sequences, for *X.carpophila* just has ITS sequences, and *X.karyophthora* from Guyana with ITS and RPB2 sequences available (Dillon et al. 2018; Vu et al. 2019). Moreover, almost half the taxa, e.g., *X.apeibae* Mont., *X.clusiae* K.F. Rodrigues, *X.duranii* San Martín & Vanoye, *X.euphorbiicola* Rehm, *X.guazumae* San Martín & J.D. Rogers, *X.heloidea* Penz. & Sacc., *X.himalayensis* Narula & Rawla, *X.jaliscoensis* San Martín, J.D. Rogers & Y.M. Ju, *X.luzonensis* Henn., *X.magnolia* J.D. Rogers, X.magnoliavar.microspora J.D. Rogers, Y.M. Ju & Whalley, *X.patrisiae* Henn., *X.psidii* J.D. Rogers & Hemmes, *X.rhizocola* (Mont.) Fr., *X.rossmanae* Y.M. Ju, J.D. Rogers, *X.terminaliae*-*bellericae* Pande & Waingankar, *X.terminaliae*-*crenulatae* Pande & Waingankar, and *X.warburgii* Henn., still have no available sequences. The current molecular study of *Xylaria* usually uses ITS, RPB2, TUB, and α-ACT (Hsieh et al. 2010; Fournier et al. 2018b; Perera et al. 2020; Ma et al. 2022), which is not so sufficient. In recent years, genome sequencing, sanger sequencing and next-generation sequencing have been used in some macrofungi groups for inferring phylogenetic relationships (Wibberg et al. 2021; Wang et al. 2023). To further understand the taxonomy and phylogeny of *Xylaria* associated with fruits and seeds, newly collected specimens from their original regions, more taxa and more DNA sequences need to be included in future study.

### ﻿Dichotomous key to species of *Xylaria* associated with fruits and seeds in China

**Table:** 

1	Ascospores pale or subhyaline	**2**
–	Ascospores brown to dark brown	**5**
2	Ascospores with a conspicuous straight germ slit	***X.theaceicola***
–	Ascospores without a germ slit or inconspicuous germ slit	**3**
3	Stromata with half- to fully exposed perithecial mounds, frequently dichotomously branched	***X.wallichii***
–	Stromata with inconspicuous perithecial mounds, unbranched in most cases	**4**
4	Stromata associated with fruits of *Magnolia* (Magnoliaceae); ascospores (13.0–)13.8–15.0(–15.6) × (3.3–) 3.6–4.0(–4.4) µm	***X.rogersii***
–	Stromata associated with fruits of *Schimanoronhae* (Theaceae); ascospores (9.5–)10.5–12.0(–13.0) × (1.6–)1.9–2.5(–3.0) µm	***X.schimicola***
5	Stromata glabrous on the fertile part	**6**
–	Stromata tomentose on the fertile part	**10**
6	Ascospores with a spiral germ slit, (12.5–)13–14(–15) × (4.8–)5.5–6.5(–6.8) µm	***X.liquidambaris***
–	Ascospores with a straight germ slit	**7**
7	Stromata associated with pericarps of fruits	**8**
–	Stromata associated with endocarps of fruits	**9**
8	Stromata associated with fruits of *Faguslongipetiolata* (Fagaceae); ascospores (11.0–)11.8–13.5(–15) × (6–)6.5–7.5(–8) µm	***Xylaria* sp.**
–	Stromata associated with fruits of *Sloanea* (Elaeocarpaceae); ascospores (9.5–)10–11.5(–12.5) × (3.5–)4–4.5(–5) µm	***X.warburgii***
9	Stromata on fallen fruits of *Reevesiaformosana* (Sterculiaceae); ascospores (8.5–)9–10.5(–11) × (4–)4.5–5.5(–6) µm	***X.reevesiae***
–	Stromata on seeds of *Crataegusoxyacantha* (Rosaceae); ascospores (10–)11–12(–12.5) × (4.5–)5.0–5.5(–6) µm	***X.oxyacanthae***
10	Stromata unbranched; ascospores (9.5–)10–11(–11.5) × (4.5–) 5–6(–6.2) µm, with an inconspicuous straight germ slit	***X.microcarpa***
–	Stromata branched	**11**
11	Stromata on buried fruits of *Aleuritesmoluccana* (Euphorbiaceae); ascospores (7.1–)7.5–9.5(–10.5) × (3–)3.5–4(–4.5) µm	***X.aleuriticola***
–	Stromata on fruits of legume pods (Fabaceae); ascospores (9.6–)10.5–13(–14) × (3.9–)4.3–5.0(–5.5) µm	***X.ianthinovelutina***

## Supplementary Material

XML Treatment for Xylaria aleuriticola

XML Treatment for Xylaria microcarpa

XML Treatment for Xylaria liquidambaris

## ﻿Additional information

### Author ORCIDs

An-Hong Zhu https://orcid.org/0000-0002-2812-8108

Zi-Kun Song https://orcid.org/0000-0001-9532-2536

Jun-Fang Wang https://orcid.org/0009-0007-1197-6008

Hao-Wen Guan https://orcid.org/0009-0000-2714-4061

Hai-Xia Ma https://orcid.org/0000-0001-6699-7454

### Data availability

Publicly available datasets were analyzed in this study. All newly generated sequences were deposited in GenBank (https://www.ncbi.nlm.nih.gov/genbank/; accessed on 26 July 2021; Table 1). All new taxa were deposited in MycoBank (https:www.mycobank.org/; accessed on 19 January 2024; MycoBank identifiers follow new taxa).
